# Polo-like-kinase 1 (PLK-1) and c-myc inhibition with the dual kinase-bromodomain inhibitor volasertib in aggressive lymphomas

**DOI:** 10.18632/oncotarget.22967

**Published:** 2017-12-06

**Authors:** Carlos Murga-Zamalloa, Avery Polk, Walter Hanel, Pinki Chowdhury, Noah Brown, Alexandra C. Hristov, Nathanael G. Bailey, Tianjiao Wang, Tycel Phillips, Sumana Devata, Pradeep Poonnen, Juan Gomez-Gelvez, Kedar V. Inamdar, Ryan A. Wilcox

**Affiliations:** ^1^ Department of Internal Medicine, Division of Hematology and Oncology, University of Michigan, Ann Arbor, MI, USA; ^2^ Department of Pathology, University of Michigan, Ann Arbor, MI, USA; ^3^ Division of Hematopathology, University of Pittsburgh, Pittsburgh, PA, USA; ^4^ Department of Pathology, Henry Ford Hospital, Detroit, MI, USA

**Keywords:** PLK-1, GATA-3, T-cell lymphoma, c-myc, volasertib

## Abstract

Survival following anthracycline-based chemotherapy remains poor among patients with most T-cell lymphoproliferative disorders. This may be attributed, at least in part, to cell-autonomous mechanisms of chemotherapy resistance observed in these lymphomas, including the loss of important tumor suppressors and the activation of signaling cascades that culminate in the expression and activation of transcription factors promoting cell growth and survival. Therefore, the identification of novel therapeutic targets is needed. In an effort to identify novel tumor dependencies, we performed a loss-of-function screen targeting ≈500 kinases and identified polo-like kinase 1 (PLK-1). This kinase has been implicated in the molecular cross-talk with important oncogenes, including c-Myc, which is itself an attractive therapeutic target in subsets of T-cell lymphomas and in high-grade (“double hit”) diffuse large B-cell lymphomas. We demonstrate that PLK-1 expression is prevalent among these aggressive lymphomas and associated with c-myc expression. Importantly, PLK-1 inhibtion with the PLK-1 inhibitor volasertib significantly reduced downstream c-myc phosphorylation and impaired BRD4 binding to the c-myc gene, thus inhibiting c-myc transcription. Therefore, volasertib led to a nearly complete loss of c-myc expression in cell lines and tumor xenografts, induced apoptosis, and thus warrants further investigation in these aggressive lymphomas.

## INTRODUCTION

Progression-free survival following anthracycline-based multi-agent chemotherapy remains poor among patients with most T-cell lymphoproliferative disorders and high-risk subsets of aggressive B-cell lymphomas. This may be attributed, at least in part, to cell-autonomous mechanisms of chemotherapy resistance observed in these lymphomas, including the loss of important tumor suppressors and the activation of signaling cascades that culminate in the activation of transcription factors that instigate the expression of genes that not only promote cell growth and survival, but also confer resistance to chemotherapy. Among cutaneous T-cell lymphomas (CTCL), for example, primary refractory disease is anticipated and treatment with multi-agent chemotherapy regimens is not recommended for most patients. While the mechanisms conferring chemotherapy resistance are likely multifactorial, including both cell-autonomous and non-cell-autonomous mechanisms, the T-cell transcription factor GATA-3 is highly expressed in many of these lymphomas and was recently shown to mediate chemotherapy resistance [12]. The most common peripheral T-cell lymphoma in North America, designated by the World Health Organization (WHO) as “not otherwise specified”, includes a distinct subset that highly expresses GATA-3 and its gene targets. Much like GATA-3^+^ CTCL, this subset of PTCL, NOS is associated with a high rate (>50%) of primary refractory disease and poor survival following conventional chemotherapy and is molecularly characterized by the expression of c-Myc related genes [1, 7, 12]. Unfortunately, median overall survival for patients with relapsed/refractory PTCL who are not candidates for high-dose therapy and autologous stem-cell transplantation remain poor, as the responses achieved with conventional and novel salvage agents are rarely complete or durable. The development of novel and effective therapies for primary refractory and chemotherapy resistant T-cell lymphomas remains an unmet clinical need. Therefore, the identification of novel therapeutic targets is needed.

## RESULTS AND DISCUSSION

In an effort to identify novel tumor dependencies in these lymphomas, we performed a loss-of-function (“Achilles heel”) screen targeting ≈500 kinases in a well characterized GATA-3^+^ CTCL cell line [1–4]. Aurora kinase A (AURKA) and its downstream target polo-like kinase 1 (PLK-1) were both identified as “hits” in this screen. These related kinases regulate mitotic entry and have been implicated in the molecular cross-talk with important oncogenes, including c-Myc [5, 6], which have been previously implicated in GATA-3^+^ T-cell lymphomas [7], and in high-risk subsets of diffuse large B-cell lymphomas. Furthermore, these kinases are frequently dysregulated in human cancers [8], and AURKA is a previously identified target in the T-cell lymphomas [9], as the selective AURKA inhibitor alisertib is associated with an overall response rate of ≈30% in these lymphomas [10]. Therefore, we sought to pharmacologically investigate the role of PLK-1 in these lymphomas using the selective PLK-1 inhibitor volasertib [11].

A library of lentiviruses expressing ∼3200 shRNAs targeting ∼501 human kinases was utilized to perform an “Achilles heel” screen in MyLa cells. This CTCL cell line was chosen as a model in this high-throughput screen as GATA-3 confers resistance to chemotherapy in a cell-autonomous manner in these cells, as observed in other cutaneous and peripheral T-cell lymphomas [1, 12]. Three independent experiments of shRNA-mediated kinase knockdown were performed, and the effect of kinase knockdown on cell viability determined. Only kinase-specific shRNAs consistently showing ≥80% inhibition of cell viability were considered for further interrogation.

**Figure 1:**
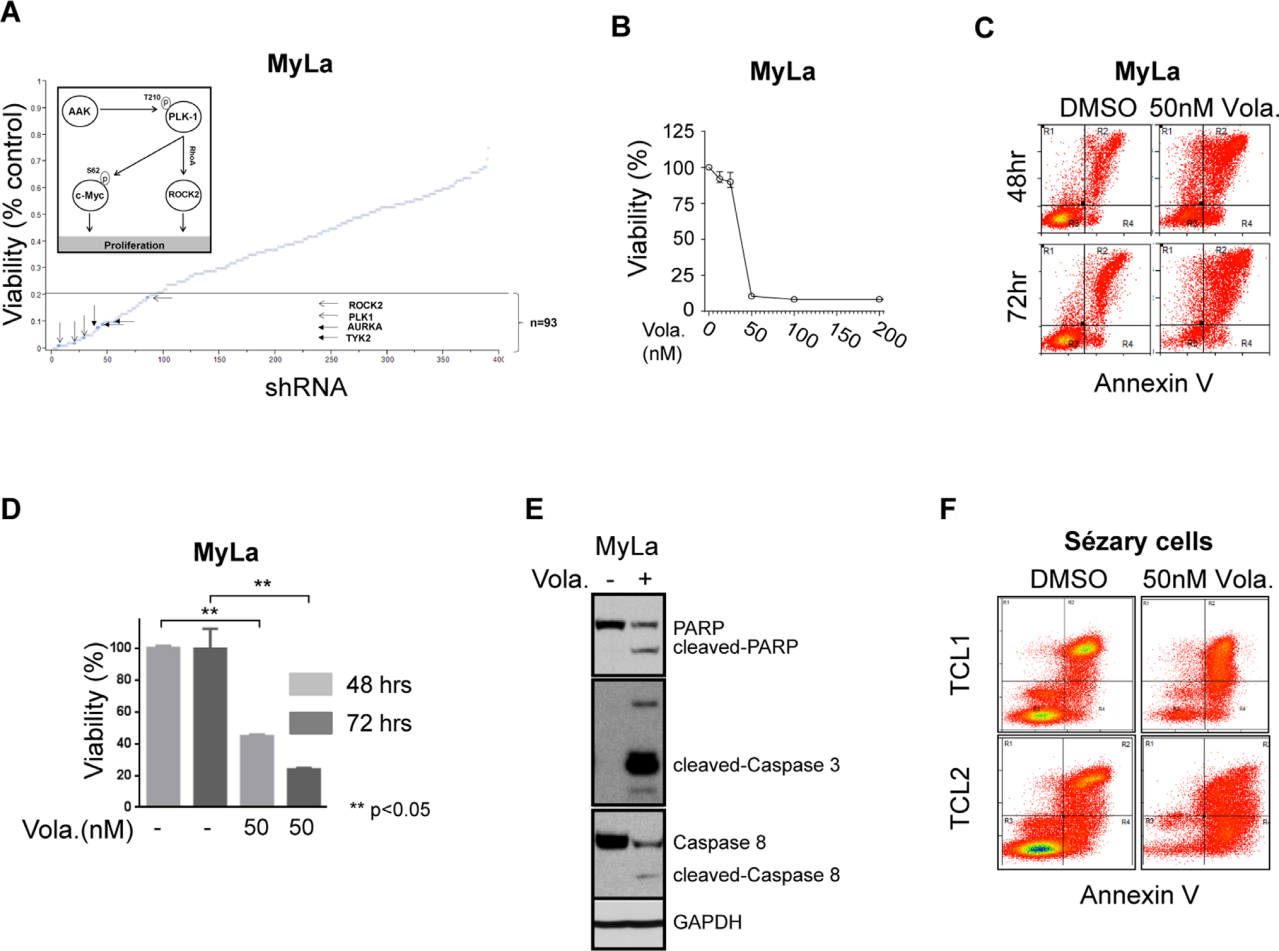
(**A**) Loss of function shRNA library screening plotted against the viability of MyLa cell line for each shRNA target. Selected kinases which decreased the viability by 80% (dotted line) are highlighted. MyLa was chosen as a model in this high-throughput screen as GATA-3 confers resistance to chemotherapy in a cell-autonomous manner in these cells, as observed in other cutaneous and peripheral T-cell lympohmas. Three independent experiments were performed and the effect of kinase knockdown on cell viability was determined by MTT assay, and normalized to non-targeting shRNA controls, as described [12]. (**B**–**E**) MyLa cells were treated with volasertib at the concentrations indicated; (B) Cell viability was measured by CellTiter Glo reagent assay from Promega Corporation, WI. (C) Apoptosis evaluated by Annexin V/propidium iodide incorporation. (D) Cell viability at 24 and 48 hours measured with CellTiter Glo assay. (E) PARP, caspase 8, and caspase 3 cleavage examined by immunoblotting. (**F**) Apoptosis following volasertib treatment was examined in two primary T-cell lymphoma samples (TCL1 and TCL2) by Annexin V/PI incorporation.

**Figure 2:**
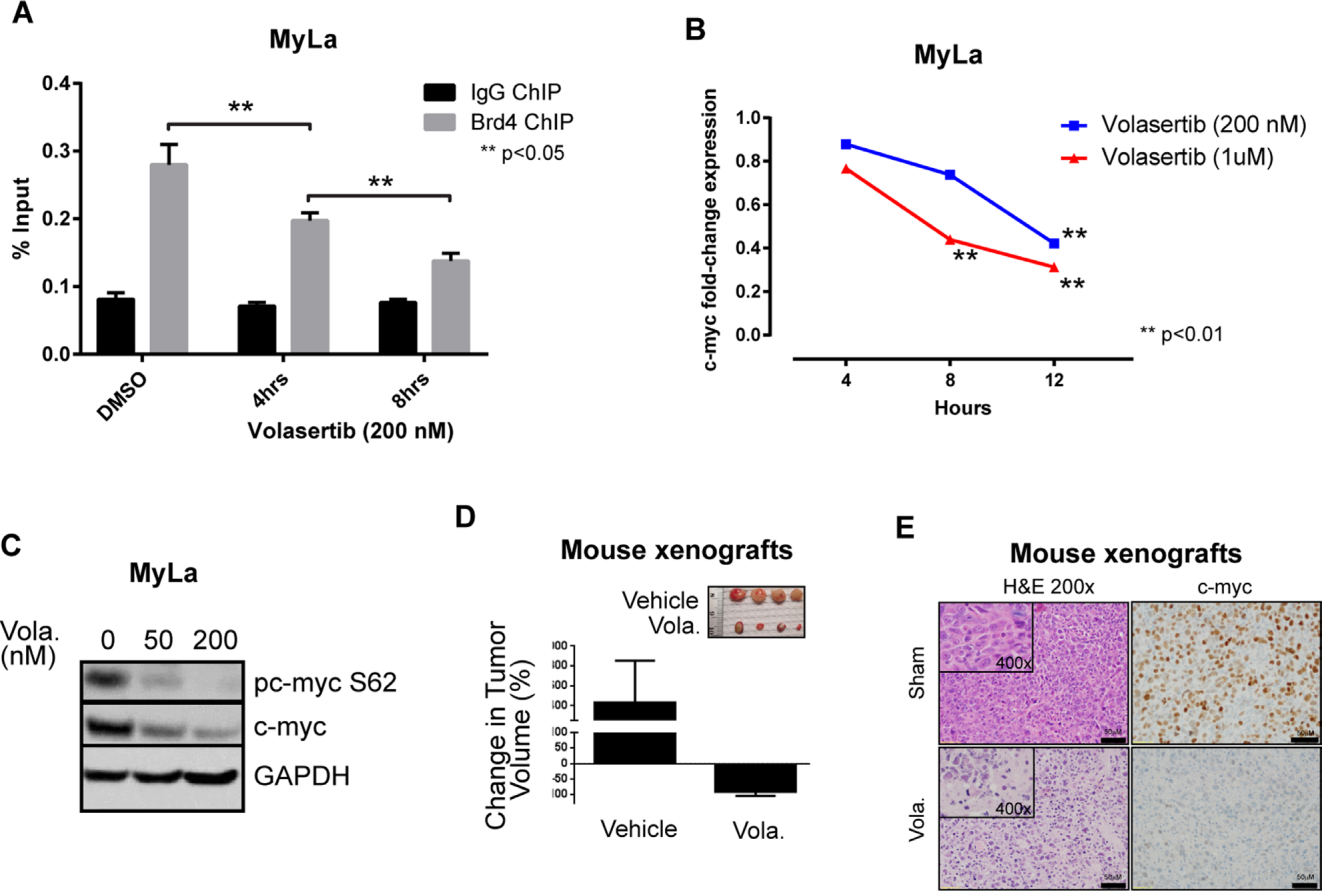
(**A**) MyLa cells were treated with volasertib and BRD4 binding to the c-myc locus examined by Chromatin immunoprecipitation assay (ChIP). (**B**) MyLa cells were treated with volasertib and c-myc gene expression was examined by quantitative real-time PCR. (**C**) C-myc phosphorylation following volasertib (Vola) treatment (overnight) in MyLa cells was examined by immunoblotting. (**D**) MyLa cells (2 × 10^6^) were injected subcutaneously in immunodeficient NSG mice. Upon tumor engraftment, volasertib (30 mg/kg) or vehicle control (DMSO) were injected intraperitoneally in 50 μL total volume (*n* = 10/group). Tumors were measured and mice humanely euthanized 4 days later. (**E**) Expression of c-myc examined by immunohistochemistry from representative samples of tumor xenografts with or without volasertib treatment.

Multiple shRNA targeting two related kinases, Aurora A (AURKA) and Polo-like kinase 1 (PLK-1), were identified in our loss-of-function screen and satisfied our a priori selection criteria (Figure 1A). PLK-1, a highly conserved serine/threonine kinase that is overexpressed in many human cancers, promotes cell cycle progression upon AURKA-dependent phosphorylation. This finding is relevant, as the AURKA inhibitor alisertib has demonstrable activity in T-cell lymphomas [10], and a PLK-1 inhibitor (volasertib) is clinically available [11]. Furthermore, our loss-of-function screen identified the RhoA associated kinase ROCK2 (Figure 1A and inset), a downstream PLK-1 substrate regulating cytokinesis via phosphorylation of regulatory myosin light chain [13]. Therefore, we sought to pharmacologically validate these findings. A significant reduction in cell viability was observed in MyLa cells treated with volasertib (IC_50_ <50 nM; Figure 1B–1D), culminating in PARP and caspase cleavage (Figure 1E), consistent with apoptotic cell death. We have previously demonstrated that T-cell receptor engagement promotes the growth and survival of malignant T cells *ex vivo*, and confers resistance to chemotherapy [12]. Therefore, primary malignant T cells (Sezary cells, *n* = 2) were purified and cultured *ex vivo* with anti-CD3/CD28 microbeads, and viability determined in volasertib (or DMSO) treated cells (Figure 1F). A significant reduction in cell viability was observed in these primary samples treated with volasertib. A recently performed screen for dual kinase-bromodomain inhibitors identified volasertib as a potent BRD4 inhibitor [14]. Therefore, volasertib may be best classified as a dual bromodomain (BRD4) and kinase (PLK-1) inhibitor. This is pertinent, as bromodomain-containing BET proteins (including BRD4) transcriptionally regulate the expression of key oncogenes, including c-myc. Furthermore, PLK-1 directly phosphorylates c-myc (serine 62, Figure 1A inset), promoting c-Myc stability. As c-myc is associated with chemotherapy resistance in aggressive T- and B-cell derived non-Hodgkin lymphomas (NHL), we sought to examine the extent to which volasertib impaired c-myc transcription and phosphorylation in MyLa cells. In accordance with its newly described role as a bromodomain inhibitor, volasertib significantly impaired BRD4 binding to a previously described c-myc enhancer (Figure 2A), leading to a commensurate inhibition in c-myc transcription (Figure 2B). PLK-1 inhibition with volasertib led to impaired c-myc phosphorylation at serine 62 (Figure 2C). Therefore, volasertib’s dual role as a BRD4 and PLK-1 inhibitor apparently converges on c-myc. To investigate this further *in vivo*, MyLa xenografts were generated in immunodeficient mice and treated with volasertib (or vehicle control). A rapid reduction in tumor volume was observed in volasertib treated mice (Figure 2D), such that only 4 (out of 10) tumors remained observable at the time of study termination (Figure 2D, inset) in volasertib-treated mice. A significant loss of c-myc expression was observed in these tumors (Figure 2E).

**Figure 3:**
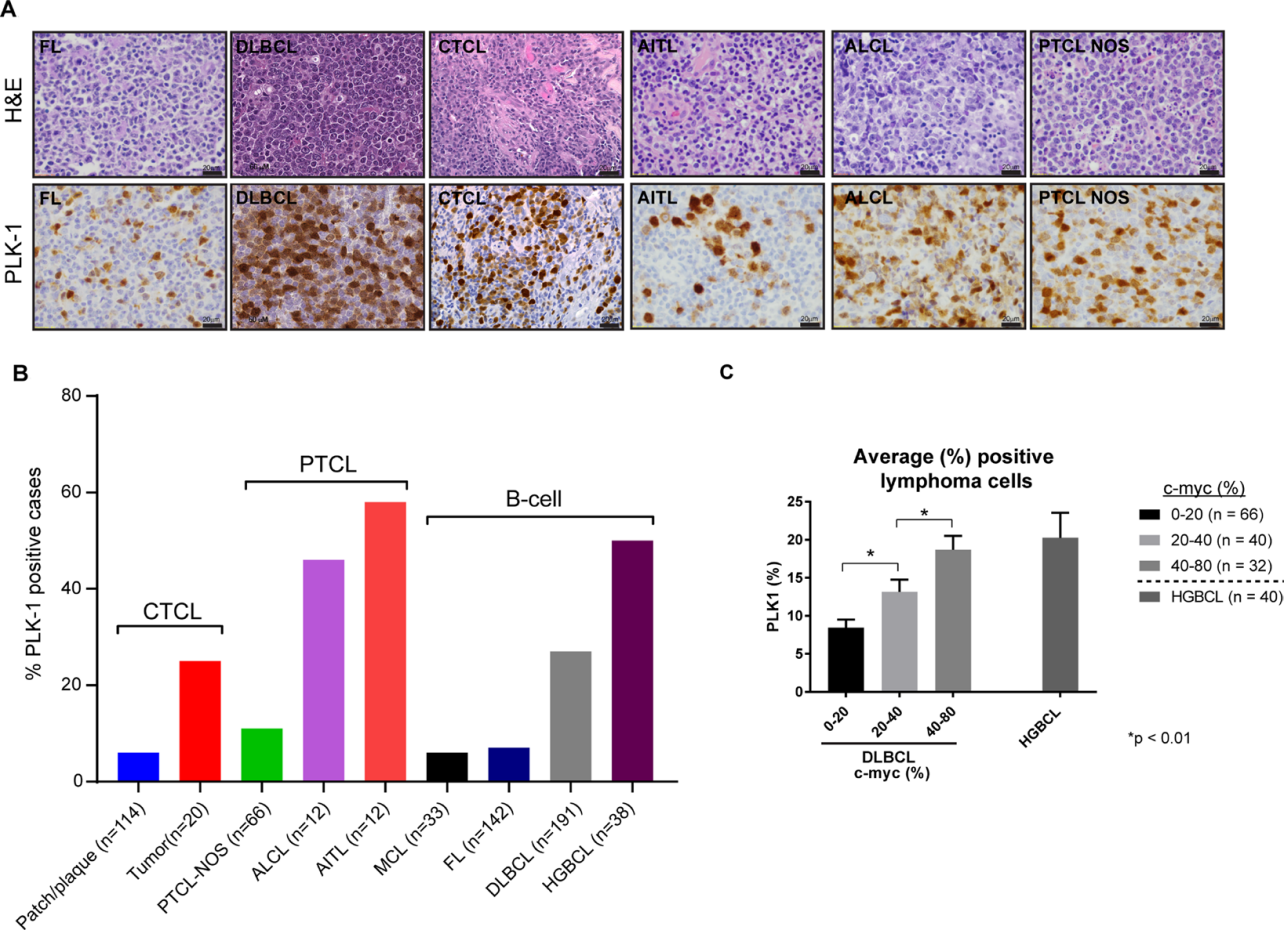
(**A**) (Left) Representative images of PLK-1 expression (lower panel) in selected cases of B-cell lymphomas (follicular lymphoma – FL and diffuse large B-cell lymphoma – DLBCL), cutaneous T-cell lymphoma (CTCL), and peripheral T-cell lymphomas (angioimmunoblastic T-cell lymphoma- AITL, anaplastic large cell lymphoma – ALCL and peripheral T-cell lymphoma non-otherwise specified – PTCL NOS). Upper panel show corresponding hematoxylin and eosin (H&E) stains for each case. (**B**) Graphic representation of the percentage of cutaneous T-cell lymphoma (CTCL), peripheral T-cell lymphomas (PTCL) and B-cell lymphomas (B-cell) positive for PLK-1. The number of nuclei expressing PLK-1 was determined and a z-score of 1, corresponding to ≥20% of nuclei, selected as the optimal cut-off. Cases with ≥40% c-myc expression were scored positive, as previously described [13]. (**C**) Graphic representation of the average percentage of lymphoma cells expressing PLK-1. DLBCL cases were divided into three groups according to the percentage of c-myc positive lymphoma cells observed by immunohistochemistry. Cases of high grade (“double hit”) B-cell lymphomas (HGBCL) are also included.

**Figure 4:**
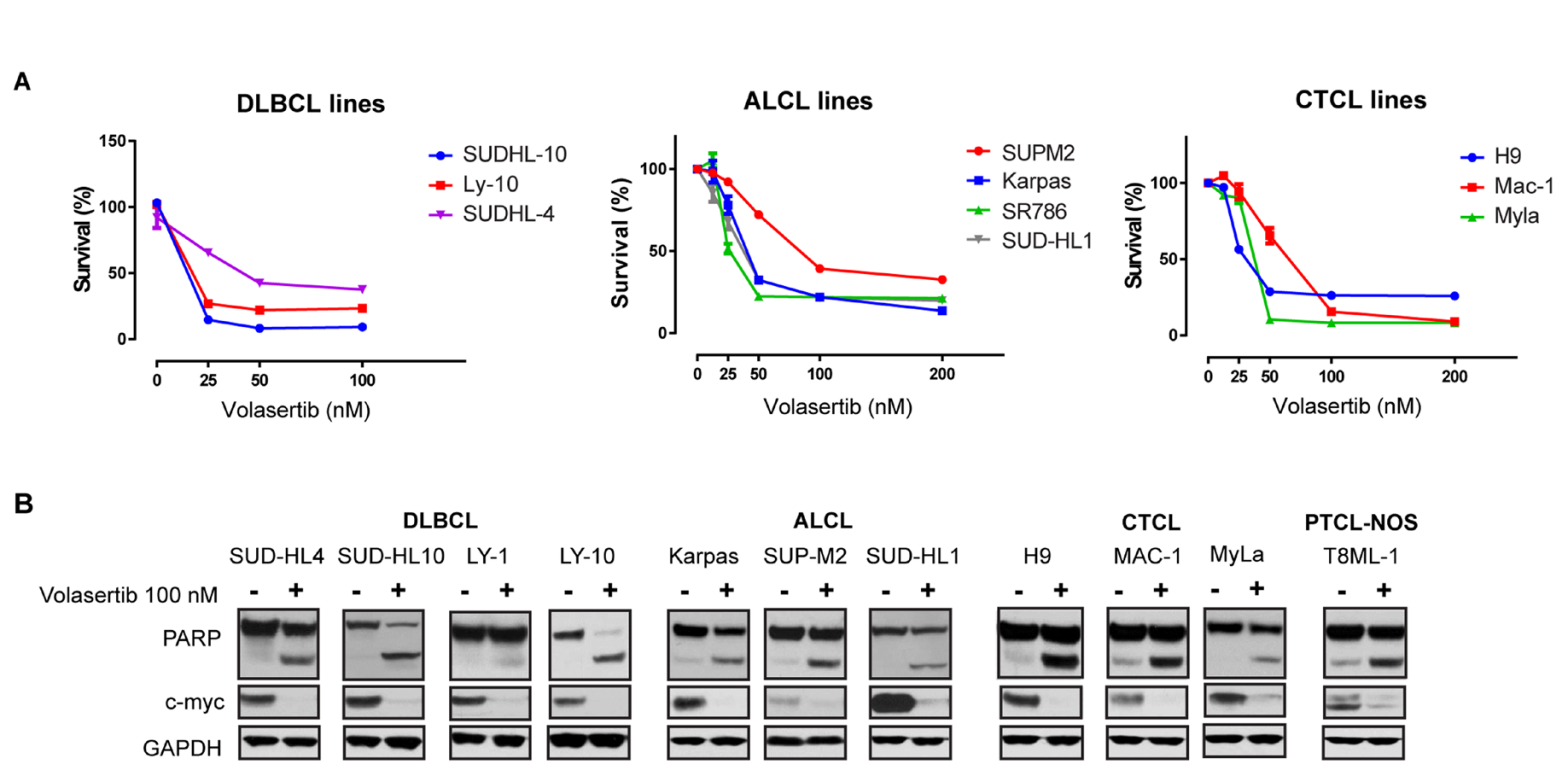
(**A**) Survival of different B-cell and T-cell lymphoma cell lines upon treatment with indicated doses of Volasertib at 72 hrs. (**B**) Treatment with volasertib (24 hours) induces PARP cleavage and decreases total c-myc expression in the indicated lymphoma cell lines. From left to right: diffuse large B-cell lymphomas (DLBCL), anaplastic large cell lymphoma (ALCL), cutaneous T-cell lymphoma (CTCL) and, peripheral T-cell lymphomas non-otherwise specified (PTCL-NOS). Protein expression was evaluated by immunoblotting.

Given these findings, we sought to examine the extent to which PLK-1 is expressed in aggressive T- and B-cell derived NHL. Among the T-cell lymphomas, 6–25% of CTCL (*n* = 134) and 11–58% of PTCL subtypes (*n* = 90) expressed PLK-1 (Figure 3A). Consistent with previous observations, among CTCL cases, PLK-1 expression was more highly expressed in tumor stage disease [15]. The increased PLK-1 expression in ALCL and AITL compared with that observed in PTCL, NOS is consistent with the results observed in a phase I study with the PLK-1 inhibitor BI-2536 [16]. In this study, both ALCL patients achieved a complete remission, whereas a single (out of 3) PTCL, NOS patients achieved a partial remission. Among B-cell lymphomas, PLK-1 expression was most prevalent in DLBCL and high grade (“double hit”) B-cell lymphomas (HGBCL) with c-myc (and Bcl-2 and/or Bcl-6) translocations, as determined by FISH (Figure 3A–3B). To further explore a correlation between PLK-1 and c-myc expression, a large cohort of DLBCL biopsies (*n* = 138) was analyzed. Our analysis demonstrated that c-myc and PLK-1 expression were strongly correlated (*R*^2^ = 0.16, *p* < 0.0001), as >80% of c-myc^+^ cases expressed PLK-1 (Figure 3C). Among “double-hit” B-cell lymphomas, 76% were positive for PLK-1 expression (*n* = 38; Figure 3C). PLK-1 significantly impaired cell viability (Figure 4A), induced PARP cleavage (Figure 4B), and significantly impaired c-myc expression in a broad spectrum of T- and B-cell lymphoma cells (Figure 4B). In summary, PLK-1 is a novel therapeutic target in aggressive T- and B-cell derived NHL, including those for which c-myc is an important oncogenic driver. Dual PLK-1 and BRD4 bromodomain inhibition warrant further investigation in these aggressive NHL.

## MATERIALS AND METHODS

### shRNA library screening

The MISSION^®^ LentiExpress^™^ Human Kinases (Sigma-Aldrich, cat#: SHX001) is a panel of lentiviruses expressing ∼3200 shRNAs targeting ∼501 human kinases. MyLa was chosen as a model in this high-throughput screen as GATA-3 confers resistance to chemotherapy in a cell-autonomous manner in these cells, as observed in other cutaneous and peripheral T-cell lympohmas [1, 12]. Three independent experiments were performed and the effect of kinase knockdown on cell viability was determined by MTT assay, as previously described, and normalized to non-targeting shRNA controls [12]. Only kinase-specific shRNAs consistently showing ≥80% inhibition of MyLa cell viability were considered for further interrogation. We elected, a priori, to prioritize these “hits” using the following criteria:1) at least one other kinase present in the same pathway was flagged and 2) a pharmacologic agent targeting a hit (or a related pathway) is clinically available.

### Antibodies and reagents

The following antibodies were used: PLK-1 (208G4), PARP (D64E10), Caspase 3 (5A1E), Caspase 8 (1C12), c-myc (D3N8F) and phospho-c-myc S62 (E1J4K), all from Cell Signaling Technologies, MA. Immunoblotting was performed, as previously described [1, 12]. Viability assays were performed using CellTiterGlo reagent and Annexin V/propidium iodide staining. Volasertib was obtained from Selleckchem.

### Nuclear/cytoplasmic cell fractionation

Nuclear fractions were extracted from cell pellets per protocol (NE-PER Nuclear & Cytoplasmic Extraction Reagents, Thermo Scientific), fixed in 16% formaldehyde, and chromatin fragmented by sonication. Diluted chromatin was immunoprecipitated using isotype control (IgG) or anti-BrD4 antibodies (Abcam) and Protein G Dynabeads. Following reversal of crosslinks and RNaseA/proteinase K digestion, immunoprecipitated DNA was purified using PCR purification kit, quantified by Nanodrop, and c-myc transcripts quantified by qPCR.

### Animal studies

Mouse studies were approved by the University Committee on Care and Use of Animals (UCUCA). Sample size for animal experiments was based on previous publications performing similar experiments, ensuring that a sufficient sample size was selected to confidently assess statistical significance. Treatment allocation was randomized, and all animals in a given experiment were included for analysis. MyLa cells (2 × 10^6^) were injected subcutaneously in immunodeficient NSG mice. Upon tumor engraftment, volasertib (30 mg/kg) or vehicle control (DMSO) were injected intraperitoneally in 50 μL total volume (*n* = 10/group). Tumors were measured and mice humanely euthanized 4 days later.

### Immunohistochemistry

Formalin fixed, paraffin sections were cut at 5 microns and rehydrated to water. Heat induced epitope retrieval was performed with FLEX TRS High pH Retrieval buffer (6.1) for 20 minutes. After peroxidase blocking, the antibody PLK1 rabbit monoclonal (208G4, Cell Signaling Technologies, MA) was applied at a dilution of 1:100 at room temperature for 60 minutes. The EnVision + Rabbit HRP System was used for detection. DAB chromagen was then applied for 10 minutes. Slides were counterstained with Harris Hematoxylin for 5 seconds and then dehydrated and coverslipped. The number of nuclei expressing PLK-1 was determined and a z-score of 1, corresponding to ≥20% of nuclei, selected as the optimal cut-off. Cases with ≥40% c-myc expression were scored positive, as previously described [13].

### Statistical analysis

Comparisons between groups were evaluated using a two-tailed Student *t*-test and all *p*-values <0.05 considered statistically significant. Event-free survival was summarized with Kaplan-Meier plots using JMP6 software and comparisons made with the log-rank test.
